# National survey of UK medical students on the perception of neurology

**DOI:** 10.1186/1472-6920-14-225

**Published:** 2014-10-21

**Authors:** Julia Pakpoor, Adam E Handel, Giulio Disanto, Richard J Davenport, Gavin Giovannoni, Sreeram V Ramagopalan, on behalf of the Association of British Neurologists

**Affiliations:** Oxford University Medical School, Medical Sciences Office, John Radcliffe Hospital, OX3 9DU Oxford, UK; Department of Physiology, Anatomy and Genetics and Medical Research Council Functional Genomics Unit, University of Oxford, Oxford, UK; Blizard Institute, Queen Mary University of London, Barts and the London School of Medicine and Dentistry, London, UK; Division of Clinical Neurosciences, Western General Hospital, Edinburgh, UK

**Keywords:** National, Survey, Medical education, Neurology, Medical student, Neurological examination

## Abstract

**Background:**

Medical students perceive neurology to be a difficult subject, a phenomenon described as “neurophobia”. Studies investigating student attitudes towards neurology have so far been limited by small sample sizes as a consequence of being conducted within a single medical school or region. We aimed to conduct the first national survey of the perception of neurology among UK medical students.

**Methods:**

A 24 question online survey was designed and distributed in the form of a web-link to all UK medical schools. Responses were collected for 10 weeks with reminders sent at 3 and 6 weeks. A prize-draw of £300 was offered upon completion of the survey.

**Results:**

2877 medical students from 25 of 31 medical schools responded**.** Students found neurology to be significantly more difficult than other specialties and were least comfortable drawing up a neurological differential diagnosis compared to other specialties (p < 0.0001 for neurology vs. each of the other specialties). Neuroanatomy was regarded as the most important factor contributing to neurology being perceived as difficult.

**Conclusions:**

The findings of the first national survey addressing this issue are consistent with previous research. The perception of neurology remains unchanged, in contrast to the rapidly changing demands of neurological care in an ageing population. Neurological examination and formulating a differential diagnosis are important skills in any medical specialty, and combatting “neurophobia” in medical students is therefore essential.

**Electronic supplementary material:**

The online version of this article (doi:10.1186/1472-6920-14-225) contains supplementary material, which is available to authorized users.

## Background

A fear of neuroscience and neurology among medical students has long been recognised and the term neurophobia was coined as long ago as 1994 [1]. Studies have suggested it is endemic among medical students and junior doctors, and associated with deficiencies in medical education [2–5]. In response, educational bodies have implemented strategies to improve the perception and experience of neurology in medical training [6, 7]. This is of particular importance given the recently modified UK medical training programme which encourages trainees to develop their career pathways early, with many students developing areas of interest as undergraduates [8].

However, the results of studies thus far have been limited by small sample sizes (with surveys typically conducted within a single medical school) and none have addressed the issue at a national level [2–5]. The data available may therefore be biased and it is unclear whether there has been a change in attitudes over time. We aimed to conduct the first national survey to determine the perception of neurology among medical students across all UK medical schools and to identify the factors influencing these views.

## Methods

A 24 question online survey was designed by medical students, neurology trainees, neurologists and neuroscience researchers from four UK medical schools. To ensure suitability and clarity of the questionnaire the survey was piloted with 10 medical students not previously involved in its design. The survey was also sent to the Association of British Neurologists for review which led to some further minor revisions. The questionnaire is available as Additional file 1. A variety of questions were used including multiple choice, short answer questions and Likert scales. Students were asked to rate neurology compared to six other specialties (gastroenterology, respiratory medicine, cardiology, geriatrics, rheumatology and endocrinology) in the following areas: difficulty learning the specialty, comfort in the relevant examination, developing a differential diagnosis and the quality of teaching received. The studied specialties and the areas explored were selected to ensure consistency with previous smaller surveys investigating the perception of neurology [2]. There was an option to provide free text responses to some questions. The final survey was distributed in the form of a web-link to UK medical students by emailing the administrative office and the undergraduate medical student society of every UK medical school with a request to distribute it to medical students. An option of entering a prize-draw of £300 was offered upon completion of the survey. The survey was set to allow only one response per computer. The survey was distributed in May 2013 and reminders were sent 3 and 6 weeks following the initial request. Responses were collected for 10 weeks from date of first distribution. The independent sample *t* test was used to compare the difference in mean score for neurology and each of the other specialties separately. Fisher’s exact test was used to assess the significance of factors influencing the likelihood of a student wishing to pursue neurology as a career. Ethical approval for the study was provided by the University of Oxford, reference MSD-IDREC-C1-2014-121.

## Results

2877 students (61.6% female and 38.4% male) from 25 out of 31 UK medical schools responded, representing approximately 7% of UK medical students. There was no notable difference in the size or location of schools which did not respond; the non-responding schools were from England, Scotland, Wales and Northern Ireland. The UK medical school training programme is typically a 5 year undergraduate degree (or 4 year graduate course) with the possible addition of an intercalated year. The median number of students responding in 25 schools was 82 (range 2–317; mode 81). The average age of respondents was 22.6 years. 83% of respondents were undergraduates, 7% were mature undergraduate students (defined as a student aged 21 or over at the start of their studies) and 10% were graduate students. Incomplete responses occurred for some questions; however, a particular pattern of non-response was not evident.

**Figure 1:**
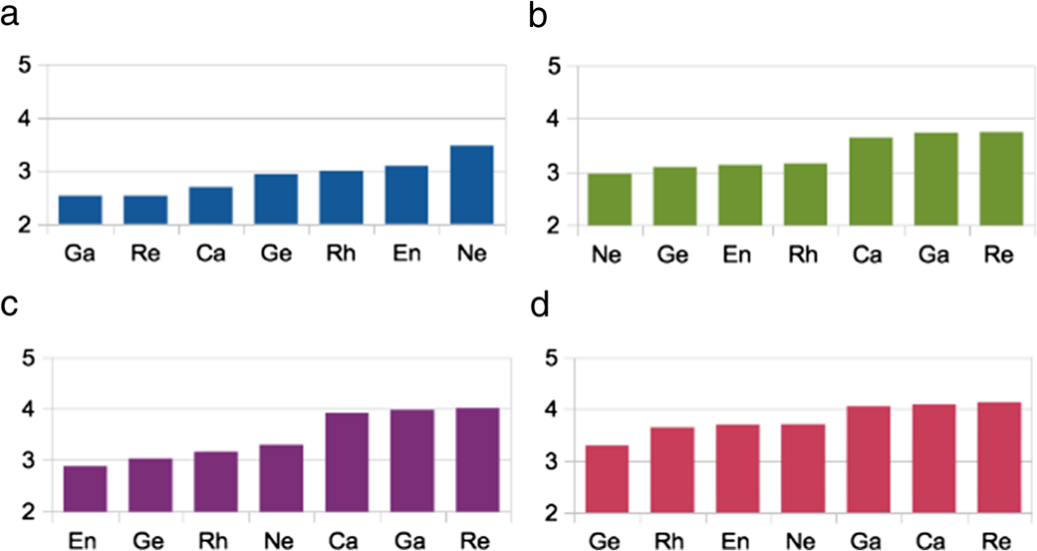
**Mean survey score results of seven medical specialties. a**. difficulty. **b**. comfort in drawing up a differential diagnosis. **c**. comfort in examination of patients. **d**. Quality of teaching. Ne = neurology, Ga = gastroenterology, Re = respiratory, Ca = cardiology, Ge = geriatrics, Rh = rheumatology and En = endocrinology. 1 = very easy/uncomfortable/poor, 2 = easy/uncomfortable/poor, 3 = moderate/satisfactory, 4 = difficult/comfortable/good, 5 = very difficult/comfortable/good.

Students found neurology to be significantly more difficult (mean score = 3.47, 95% confidence interval (95% CI) = 3.43 to 3.51) than any other specialty (p < 0.0001 for neurology vs. each of the other specialties) (Figure 1a) and also reported being the least comfortable in drawing up a differential diagnosis from a presentation of neurological symptoms (mean score = 2.96, 95% CI = 2.92 to 3.00) compared to other specialties (p < 0.0001 for neurology vs. each of the other specialties) (Figure 1b). Neuroanatomy was identified as the biggest factor making neurology difficult with 70% rating it a large or very large contributor to the level of difficulty, followed by basic neuroscience (45%) and lack of diagnostic certainty (40%). The level of comfort in examining neurological patients and the quality of teaching received in neurology was rated higher than endocrinology, geriatrics and rheumatology (Figure 1c-d). Upon including only the 1461 respondents who had at the time of the survey completed both the pre-clinical neuroscience and clinical neurology components of their course, students still found neurology to be significantly more difficult (mean score = 3.37, 95% CI = 3.31 to 3.43) than any other specialty (p < 0.0001 for neurology vs. each of the other specialties). These students were also significantly less comfortable in drawing up a differential diagnosis from a presentation of neurological symptoms (mean score = 3.22, 95% CI = 3.17 to 3.27) compared to all other specialties (p < 0.0001 for neurology vs. each of the other specialties), except for endocrinology (p = 0.0712). In the “open comments” section it was clear that students felt that there was a lack of integration between pre-clinical neuroscience and clinical components of neurology training, as well as an insufficient length of time dedicated to neurology in the medical course, which in some schools is not a distinct clinical rotation but integrated into other medical attachments.

**Figure 2:**
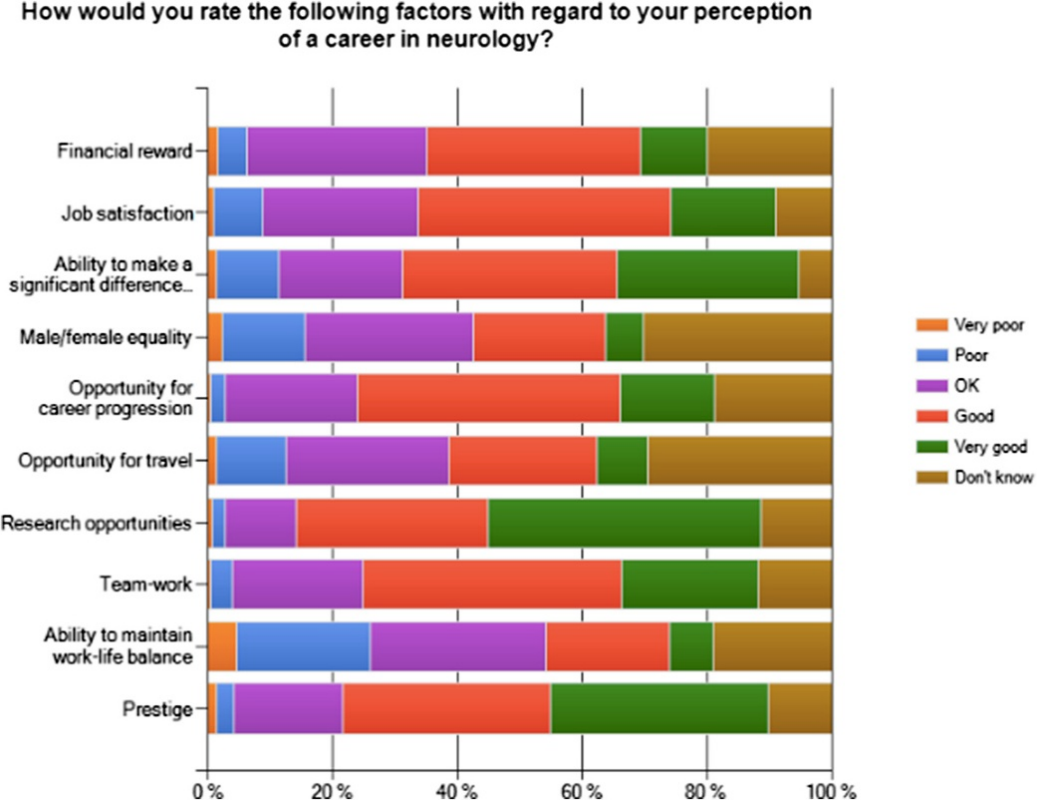
**Student perceptions of a career in neurology.**

Regarding the possibility of a career in neurology, students ranked neurology higher than rheumatology, endocrinology and geriatrics as a prospective career. Respondents also considered it to be associated with good or very good research opportunities (75%), prestige (68%) and the ability to make a significant difference to patients’ lives (64%). Job satisfaction and ability to make a significant difference to patients’ lives were the most likely factors to persuade students to pursue a career in neurology (32% and 30% respectively). On the contrary, 26% thought there was a poor or very poor ability to maintain work-life balance, which was further shown to be the most likely factor to dissuade students from pursuing a career in neurology (43%). These data are presented in Figure 2.

The following factors were associated with a significantly increased likelihood of pursuing neurology as a career: being male (p < 0.0001), personal experience caring for a relative or friend suffering from a neurological disorder (p = 0.04) or caring for someone suffering from a neurological disorder through volunteer work in a healthcare environment (p < 0.0001).

Among students who had completed both the pre-clinical neuroscience and clinical neurology components of their course, 35% of students felt that the amount of planned neurology teaching was too little and more/improved bedside teaching was ranked as the factor which would most improve the neurology training in medical school. Further, 42% reported having not had the opportunity to receive additional neurology teaching beyond the course curriculum, 27% had not met a neurologist who inspired them, 26% did not feel confident they knew what neurologists do and 20% reported having not had the opportunity to carry out a clinical placement in neurology.

## Discussion

Our study is the largest and the first national study to investigate perceptions of neurology by medical students. We have confirmed previous findings that students consider neurology to be the most difficulty specialty to learn and the one in which they feel the least comfortable establishing a differential diagnosis. Although we have only surveyed students, other work has indicated that neurophobia persists after qualification [2, 7]. A study of general practitioners, who are often the sole providers of care for common neurological conditions such as migraine, reported a lack of confidence in addressing neurological complaints, likely to result in increased referrals and demand on specialist care [9, 10]. The fact that neuroanatomy and learning basic neurosciences were identified as the most important driving factors of the difficulty of neurology highlights the need to reduce the substantial time gap between basic neuroscience and clinical teaching at many medical schools and to adopt a more integrated structure. Resources through which to teach and enable an understanding of neuroanatomy may be scarce and likely to be helped by the use of online resources. Similarly, the use of available online videos and demonstrations are likely to aid teaching of the neurological examination. An American study investigating alternative methods of teaching neurology found that 6 years following the implementation of an e-textbook student satisfaction had risen, and it was identified to be an effective tool to aid the teaching of neurology [11].

Medical schools should ensure that they assess the perception of neurology among their students, collect feedback and subsequently assess the effectiveness of any interventions over time. The widespread scale of neurophobia warrants a national initiative and we propose the establishment of a massive open online course for large-scale participation aimed at teaching functional neuroanatomy around the neurological examination.

Further, our findings demonstrate that the perception of neurology across more than a decade has remained unchanged, in sharp contrast to the rapidly changing demands of neurological care. The growing social and financial burden of an ageing population with chronic neurological diseases, particularly neurodegenerative disorders, and a relative shortage of neurologists in the UK has highlighted the need for a multidisciplinary approach to their care [12, 13]. Management of neurological diseases will be an unavoidable reality for most future doctors and focusing on the effective development of neurological skills is likely to be a cost-effective measure in providing optimal early care and appropriate referral.

The perceived level of difficulty of neurology is not reflected by the interest in the subject. This is encouraging and in line with findings from previous work, suggesting that students want to grasp the subject and may be motivated to work hard at it [2, 4, 14]. The finding that one in four students thought there was a poor or very poor ability to maintain work-life balance in neurology is perhaps surprising, and a potential misconception which could be addressed through career talks.

Limitations of this study include the response rate (as despite being the largest survey, only 7% of medical students responded) and the fact that we cannot exclude the possibility of institutional bias (as response rates were not equal across institutions), responder bias and acquiescence bias (a tendency to respond positively to survey questions, which we tried to dissipate as much as possible through options including for example “don't know” and “neither likely or unlikely”).

## Conclusions

In conclusion, we report that students perceive neurology to be the most difficult specialty to learn and in which to formulate a differential diagnosis. We encourage neurologists and course organisers to work towards greater understanding of neuroanatomy and basic neuroscience through integration of pre-clinical and clinical neurology teaching, for example by increasing case-based/bedside teaching and ensuring teaching remains relevant and focused on the most important principles. Ensuring that medical students are comfortable with the neurological examination and diagnosis is a necessary priority not only for medical students wishing to pursue a career in neurology, but for many other specialties, particularly primary care.

## Electronic supplementary material

Additional file 1: **Survey questionnaire.** (PDF 914 KB)
