# Supplementary material for: National survey of UK medical students on the perception of neurology
Source: BMC Med Educ. 2014 Oct 21;14:225. doi: 10.1186/1472-6920-14-225 (PMC4295337; doi:10.1186/1472-6920-14-225)
Supplement: Supplementary file 1 — Additional file 1: Survey questionnaire. (PDF 914 KB) [file 12909_2014_1101_MOESM1_ESM.pdf]

National Survey of Medical Students on the Perception of Neurology

1.

1. What is your age?

[▼]

2. What is your gender?

- ( ) Female
- (●) Male

3. Which medical school do you attend?

[▼]

Other (please specify)

[ ]

4. What type of medical course are you on?

- ( ) Undergraduate
- ( ) Mature undergraduate
- ( ) Graduate

5. How long is your medical school course (including the intercalated year if applicable)?

[▼]

6. What is your expected year of graduation?

[▼]

Other (please specify)

[ ]

7. How difficult do you think it is for students to learn about the following areas of medicine?

	Very difficult	Difficult	Moderate	Easy	Very easy	No teaching given
Cardiology	( )	( )	( )	( )	( )	( )
Gastroenterology	(●)	( )	( )	( )	( )	( )
Respiratory medicine	( )	( )	( )	( )	( )	( )
Neurology	( )	( )	( )	( )	( )	( )
Endocrinology	( )	( )	( )	( )	( )	( )
Rheumatology	( )	( )	( )	( )	( )	( )
Geriatrics	( )	( )	( )	( )	( )	( )

8. How comfortable are you in your ability to examine patients with disorders related to the following medical areas?

	Very uncomfortable	Uncomfortable	Moderately	Comfortable	Very comfortable	No teaching given
Cardiology	○	○	○	○	○	○
Gastroenterology	○	○	○	○	○	○
Respiratory medicine	○	○	○	○	○	○
Neurology	○	○	○	○	○	○
Endocrinology	○	○	○	○	○	○
Rheumatology	○	○	○	○	○	○
Geriatrics	○	○	○	○	○	○

9. How comfortable are you in drawing up a differential diagnosis for patients presenting with symptoms in the following area

	Very uncomfortable	Uncomfortable	Moderately	Comfortable	Very comfortable	No teaching given
Cardiology	○	○	○	○	○	○
Gastroenterology	○	○	○	○	○	○
Respiratory medicine	○	○	○	○	○	○
Neurology	○	○	○	○	○	○
Endocrinology	○	○	○	○	○	○
Rheumatology	○	○	○	○	○	○
Geriatrics	○	○	○	○	○	○

10. How would you rate the quality of the teaching you have received in the following medical areas?

	Very poor	Poor	Satisfactory	Good	Very good	No teaching given
Cardiology	○	○	○	○	○	○
Gastroenterology	○	○	○	○	○	○
Respiratory medicine	○	○	○	○	○	○
Neurology	○	○	○	○	○	○
Endocrinology	○	○	○	○	○	○
Rheumatology	○	○	○	○	○	○
Geriatrics	○	○	○	○	○	○

11. Please rank the following specialties by how likely you are to want to pursue a career in each specialty:

☐	Cardiology	☐ N/A
☐	Gastroenterology	☐ N/A
☐	Respiratory medicine	☐ N/A
☐	Neurology	☐ N/A
☐	Endocrinology	☐ N/A
☐	Rheumatology	☐ N/A
☐	Geriatrics	☐ N/A
☐	Not interested in pursuing any of the above	☐ N/A

2.

12. Have you had any personal experience (outside of your medical course) of caring for someone with a neurological disorder?

	Yes	No
Cared for a relative or a friend suffering from a neurological disorder	◯	◯
Cared for someone suffering from a neurological disorder through volunteer work in a healthcare environment	◯	◯
Cared for someone suffering from a neurological disorder through paid work	◯	◯

Other (please specify)

13. To what extent have you covered neurology teaching so far in your course (if "not at all", please continue to the next page)

- ◯ Not at all
- ◯ Pre-clinical neuroscience only
- ◯ Clinical neurology only
- ◯ Both pre-clinical neuroscience and clinical neurology

14. Do you think the amount of planned teaching in neurology you have received was: Too little/about right/too much

◯ Too little ◯ About right ◯ Too much

Other (please specify)

15. Have you had the opportunity so far in your medical course to do the following:

	Opportunity available and taken	Opportunity available but not taken	Opportunity not available
The opportunity to carry out a research project in a topic related to neurology	○	○	○
The opportunity to receive additional neurology teaching beyond course curriculum	○	○	○
The opportunity to discuss neurological cases through problem-based learning or case-presentations	○	○	○
Carry out a clinical placement in neurology	○	○	○
Meet a neurologist who has inspired you	○	○	○

16. To what extent do you think the following factors contribute to neurology being perceived as a difficult subject?

	No contribution	Small contribution	Moderate contribution	Large contribution	Very large contribution	Don't know
Neuroanatomy	○	○	○	○	○	○
Learning basic neuroscience	○	○	○	○	○	○
The clinical neurological examination	○	○	○	○	○	○
Lack of diagnostic certainty	○	○	○	○	○	○
Poor quality/lack of teaching	○	○	○	○	○	○
Lack of clinical exposure to neurological patients	○	○	○	○	○	○

Other (please specify)

[ ]

17. Please rank the following in the order in which they would most improve the neurology training at your medical school?

	1	2	3	4	5	N/A
More/improved online learning resources	○	○	○	○	○	○
More/improved text books	○	○	○	○	○	○
More/improved lectures	○	○	○	○	○	○
More/improved bedside teaching	○	○	○	○	○	○
More/improved peer discussions	○	○	○	○	○	○

Other (please specify)

[ ]

3.

18. How would you rate the following factors with regard to your perception of a career in neurology?

	Very poor	Poor	OK	Good	Very good	Don't know
Financial reward	◯	◯	◯	◯	◯	◯
Job satisfaction	◯	◯	◯	◯	◯	◯
Ability to make a significant difference to patient's lives	◯	◯	◯	◯	◯	◯
Male/female equality	◯	◯	◯	◯	◯	◯
Opportunity for career progression	◯	◯	◯	◯	◯	◯
Opportunity for travel	◯	◯	◯	◯	◯	◯
Research opportunities	◯	◯	◯	◯	◯	◯
Team-work	◯	◯	◯	◯	◯	◯
Ability to maintain work-life balance	◯	◯	◯	◯	◯	◯
Prestige	◯	◯	◯	◯	◯	◯

19. Which one factor is most likely to dissuade you from pursuing a career in neurology?

20. Which one factor is most likely to persuade you to pursue a career in neurology?

4.

21. Do you feel you have had sufficient opportunity to get involved in activities related to neurology outside your medical course (such as invited speakers, conferences, journal clubs etc)?

- ○ Strongly disagree
- ○ Disagree
- ○ Neither agree nor disagree
- ○ Agree
- ○ Strongly agree
- ○ Don't know

22. Do you feel confident that you know what neurologists do?

- ○ Strongly disagree
- ○ Disagree
- ○ Neither agree nor disagree
- ○ Agree
- ○ Strongly agree

23. How likely are you to pursue a career in neurology?

- ○ Definitely not
- ● Unlikely
- ○ Neither likely nor unlikely
- ○ Likely
- ○ Definitely will
- ○ Don't know

24. We welcome any other comments you may have on your perception and experience of neurology and how this can be improved:

25. Thank you for taking part in this survey.

All information in this survey is treated anonymously. However, in order to take part in our prize-draw of £300 please enter your email here. Your email will ONLY be used in the prize-draw and will be discarded as soon as the prize-draw is complete. Email address (optional):
